# Elucidating the Activation Mechanism of AMPK by Direct Pan-Activator PF-739

**DOI:** 10.3389/fmolb.2021.760026

**Published:** 2021-11-05

**Authors:** Elnaz Aledavood, Aria Gheeraert, Alessia Forte, Laurent Vuillon, Ivan Rivalta, F. Javier Luque, Carolina Estarellas

**Affiliations:** ^1^ Department of Nutrition, Food Science and Gastronomy, Faculty of Pharmacy and Food Sciences, and Institute of Theoretical and Computational Chemistry (IQTCUB), University of Barcelona, Barcelona, Spain; ^2^ Dipartimento di Chimica Industriale “Toso Montanari” Università di Bologna, Bologna, Italy; ^3^ LAMA, University of Savoie Mont Blanc, CNRS, LAMA, Le Bourget du Lac, France; ^4^ Université de Lyon, École Normale Supérieure de Lyon, CNRS UMR 5182, Laboratoire de Chimie, Lyon, France; ^5^ Institute of Biomedicine (IBUB), University of Barcelona, Barcelona, Spain

**Keywords:** AMPK, protein dynamic, protein activation mechanism, pan-activator, isoform selectivity, molecular dynamics simulation

## Abstract

Adenosine monophosphate-activated protein kinase (AMPK) is a key energy sensor regulating the cell metabolism in response to energy supply and demand. The evolutionary adaptation of AMPK to different tissues is accomplished through the expression of distinct isoforms that can form up to 12 heterotrimeric complexes, which exhibit notable differences in the sensitivity to direct activators. To comprehend the molecular factors of the activation mechanism of AMPK, we have assessed the changes in the structural and dynamical properties of β1- and β2-containing AMPK complexes formed upon binding to the pan-activator PF-739. The analysis revealed the molecular basis of the PF-739-mediated activation of AMPK and enabled us to identify distinctive features that may justify the slightly higher affinity towards the β1−isoform, such as the β1−Asn111 to β2−Asp111 substitution, which seems to be critical for modulating the dynamical sensitivity of β1- and β2 isoforms. The results are valuable in the design of selective activators to improve the tissue specificity of therapeutic treatment.

## Introduction

AMP-activated protein kinase (AMPK) is a Ser/Thr protein kinase with a key role as a sensor in cellular energy homeostasis (Xiao et al., 2011). Upon activation, AMPK increases the levels of ATP, favoring the reduction of anabolic pathways and up-regulation of catabolic pathways. Due to its critical role in cell metabolism, AMPK is implicated in numerous metabolic disorders such as type 2 diabetes, cardiovascular diseases, and obesity (Carling, 2017). However, one of the most interesting aspects of this enzyme comes from the different tissue distribution that is directly related to its structural complexity. AMPK is a heterotrimeric complex consisting of a catalytic α-subunit and two regulatory subunits, namely β and γ. Each subunit can be found in different isoforms, involving two for α (α1, α2), two for β (β1, β2), and three for γ (γ1, γ2, γ3) (Calabrese et al., 2014). The N-terminus of the α catalytic subunit contains a kinase domain, while its C-terminus is needed for the formation of the complex with the other subunits. The β-subunit has a central carbohydrate-binding module (CBM) that mediates AMPK interaction with glycogen, and the C-terminal region acts as a scaffold for the heterotrimeric assembly. Finally, the γ-subunit has four tandem repeats of the cystathionine β-synthase (CBS) domain, forming up to four potential nucleotide binding sites although only sites 1, 3 and 4 can really bind them (Scott et al., 2004; Scott et al., 2008; Carling et al., 2012).

**FIGURE 1:**
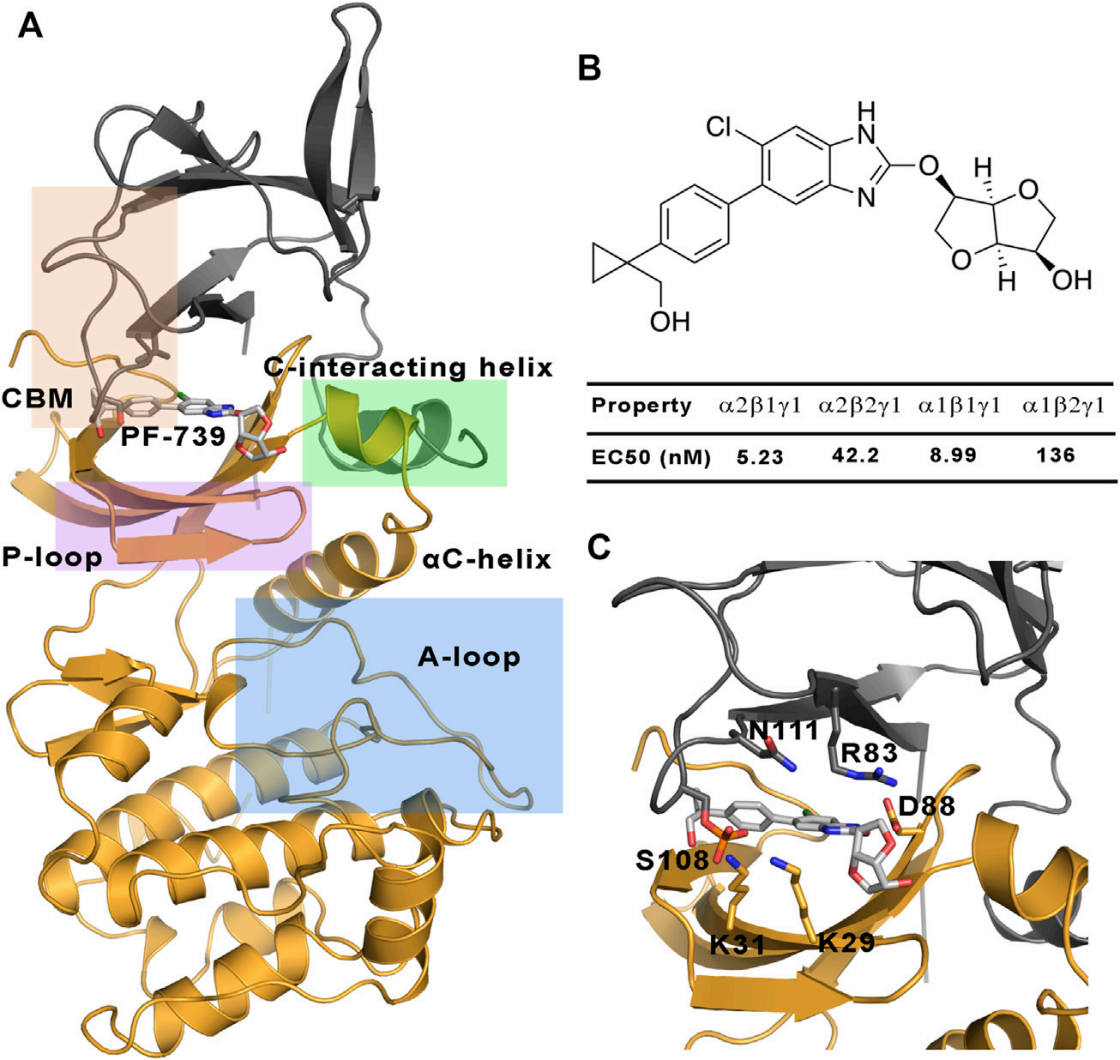
**(A)** Representation of the system selected for the study of the direct activation mechanism, which is composed of α2 **(yellow cartoon)** and β1-/β2-subunits **(grey cartoons)**. The most important regions of these subunits are highlighted: P-loop **(purple)**, activation loop (A-loop, **cyan**), CBM domain **(orange)** and C-interacting helix **(green)**. **(B)** Chemical structure of PF-739, together with the experimental data obtained in Cokorinos et al. (2017) for the half maximal effective concentration (EC_50_). **(C)** The activator bound to the ADaM site, with selected residues of the ADaM site shown as sticks.

AMPK is finely regulated by different mechanisms (Mahlapuu et al., 2004). An allosteric activation involves the phosphorylation of α2-Thr172 in the activation loop of the kinase domain by upstream kinases such as LKB1 and CaMKKb, together with the binding of AMP to the CBS domain in the γ−subunit. The active AMPK complex can thus respond to subtle fluctuations in the AMP/ATP ratio, it being several thousand-fold more active (Carling et al., 2012; Chen et al., 2012; Willows et al., 2017). On the other side, AMPK can also be indirectly activated by compounds such as metformin, phenformin and oligomycin (Vazquez-Martin et al., 2012), which are able to increase the intracellular levels of AMP. However, much interest is focused on the understanding of the direct activation mechanism of AMPK by small organic molecules. The first reported direct activator was the thienopyridone drug A-769662 (Cool et al., 2006), which is bound to a cavity located at the interface between the CBM domain of the β-subunit and the kinase domain of the α-subunit, namely the allosteric drug and metabolite (ADaM) site (Langendorf and Kemp, 2015). One of the main features of the direct activation is that this kind of activation is independent of the Thr172 phosphorylation, while it is enhanced by phosphorylation of Ser108 in the CBM domain of the β-subunit, increasing the AMPK activity by >90-fold (Hardie, 2014). Since then, a lot of efforts have been invested in obtaining direct AMPK activators, which in some cases exhibit a marked isoform selectivity (Olivier et al., 2018), while in other cases no significant selectivity is observed towards specific subunit isoforms. The isoform selectivity is relevant for the tissue distribution of the AMPK complexes. While α1, β1 and γ1 have low tissue specificity, α2 is basically found in the heart and skeletal muscle, β2 in the skeletal muscle and γ2 is mainly found in the heart muscle, and γ3 is found in the skeletal muscle (Uhlén et al., 2015; Human Protein Atlas (2021, 2021). The tissue specificity is related to the specific function of AMPK in these tissues, and therefore all the isoforms in the skeletal muscle have an important role in the glucose uptake, making AMPK a promising target for diabetes type 2 disease. In the last years an increasing effort has been devoted to design tissue-specific direct AMPK activators. As an example, the SC4 small-molecule, which was designed to increase the selectivity towards the α-subunit (being more selective for the α2-isoform) (Ngoei et al., 2018), can activate both β1- and β2-containing AMPK complexes, although a slightly higher activation is observed for the β1-isoform. Other interesting examples are the pan-activators PF-739, which is able to activate both α2β1γ1 and α2β2γ1 (Figure 1), and MK-8722 which can activate the 12 heterotrimeric AMPK complexes (Myers et al., 2017). Regarding the selectivity of β-isoform, although the half maximal effective concentration (EC_50_) determined for PF-739 and the binding affinity measurements for MK-8722 shows that they still exhibit a larger affinity for the β1-containing isoforms, they are the most potent activators of β2 complexes reported up to date (Cokorinos et al., 2017). However, it is still necessary to achieve a higher specificity to avoid off-tissue target effects. Accordingly, understanding of the molecular factors that favor the binding to specific isoforms is an outstanding issue.

In our previous works (Aledavood et al., 2019; Aledavood et al., 2021), we have studied the molecular factors that determine the selective activation of β1- and β2-containing AMPK complexes formed with A-769662 and SC4. We have hypothesized that the change of β1-Asn111 by β2-Asp111 could be a key factor in mediating the distinctive “mechanical” sensitivity of AMPK complexes to these activators. Here, we extent this analysis to the pan-activator PF-739 with the aim to examine how the binding of this compound affects the dynamical response of AMPK considering the trends disclosed for A-769662 and SC4. At this point, it is worth noting that while A-769662 is selective for β1-containing complexes, SC4 exhibits a mild preference for this isoform, a trend which was attributed to the presence of the carboxylate group present in the chemical structure of this activator. In contrast, PF-739 is a neutral compound, which suggests that other chemical features might also regulate the mild preference for binding to β1-containing AMPK complexes. Understanding the role of the factors that regulate the mechanical response of AMPK could thus be valuable for the tailored design of isoform-adapted pharmacophores useful in the search of selective direct activators. With this aim in mind, we have carried out extensive molecular dynamic simulations (MD) and network analysis to examine the differential trends in structural, dynamical and interaction patters emerging for AMPK complexes with PF-739.

## Results and Discussion

MD simulations were run to assess the structural and dynamics properties of the AMPK complexes formed by the α2-isoform bound to either β1- or β2- subunits. The neglect of the γ subunit in the simulated systems obeys two main motivations. First, following a divide-and-conquer strategy, this permits to focus the conformational sampling of the activator-induced changes on the ADaM site, which is shaped by residues in α and β subunits. Second, the adoption of these systems permits a direct comparison with the results obtained previously for the complexes formed with A-769662 and SC4 (Human Protein Atlas (2021, 2021; Ngoei et al., 2018). Accordingly, this study is focused on the conformational ensemble collected for the apo species of α2β1 and α2β2 systems, the corresponding complexes formed with PF-739 (holo species), and finally the complexes formed with both PF-739 and ATP molecule (holo+ATP), the latter being located in the ATP-binding site within the kinase domain of the α−subunit. For each system (apo, holo, and holo+ATP), the analysis involves the conformational ensemble explored in three independent replicas (1 μs/replica), leading to a total simulation time of 6 μs for the apo species and 12 μs for the ligand-bound complexes.

### Structural Analysis of AMPK Complexes

**FIGURE 2:**
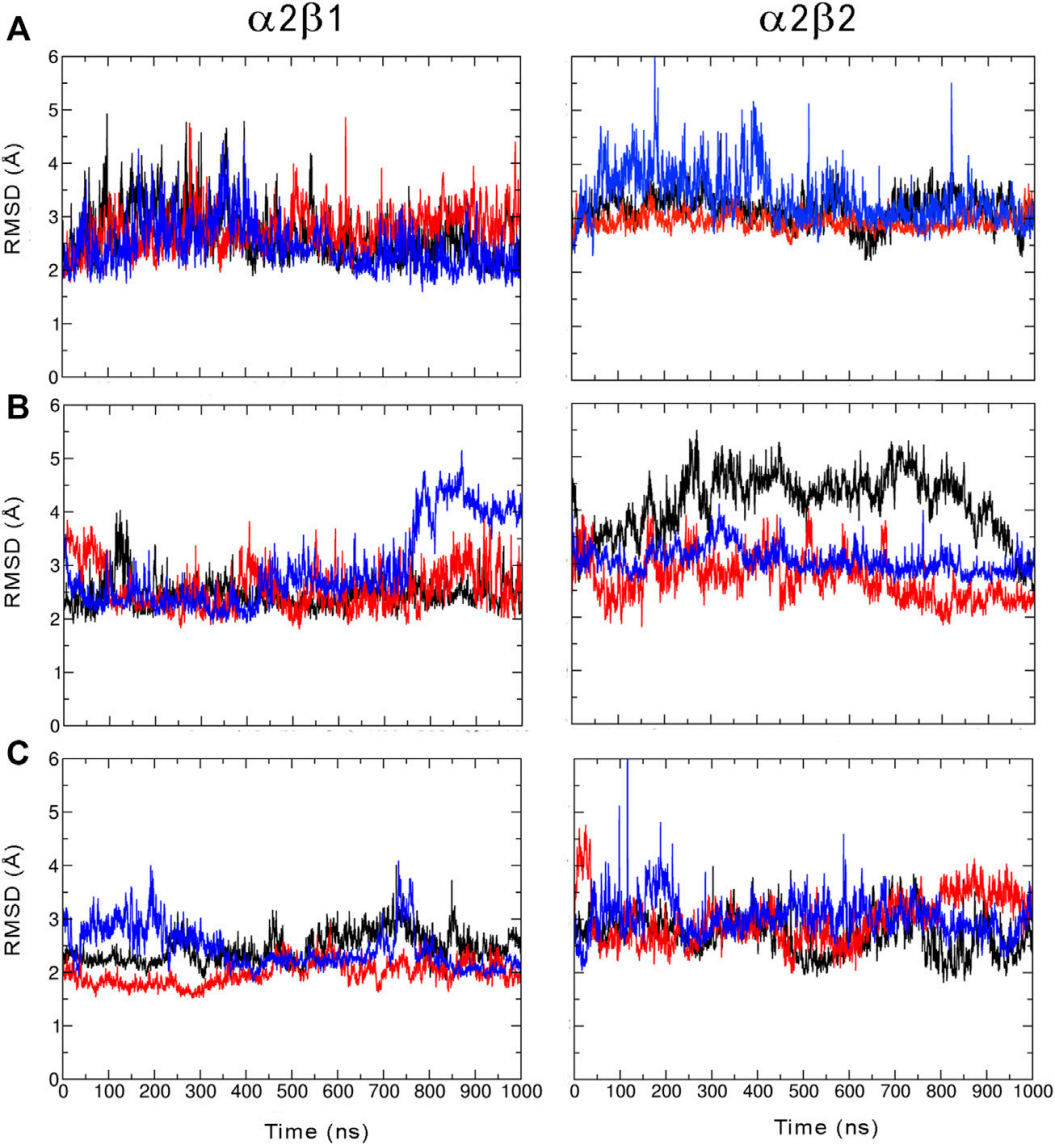
Root mean squared deviation (RMSD, Å) determined for the protein backbone along the three 1 µs MD simulations run for the **(A)** apo, **(B)** holo and **(C)** holo+ATP species of AMPK isoforms α2β1 and α2β2 bound to PF-739 (each replica is shown in black, blue and red, respectively). For each analysis the reference structure used corresponds to the energy-minimized average structure of the holo+ATP sampled in the last 200 ns of the three independent MD simulations.

We have examined the effect of PF-739 binding to the ADaM site (holo structures), and the simultaneous presence of PF-739 and ATP in both ADaM and ATP-binding sites (holo+ATP structures) on the global structural conformation of apo α2β1 and α2β2 by means of the root mean square deviation (RMSD) of the protein backbone along the corresponding 1 μs simulations (Figure 2). The RMSD was determined using the average structure of the holo+ATP species sampled in the last 200 ns of the three independent replicas run for either α2β1 or α2β2 species as reference. For the holo+ATP systems there is a high structural resemblance for all the replicas, as noted in the small fluctuations of the RMSD profiles (Figure 2C), which agrees with the preservation of the overall protein fold upon binding of both PF-739 and ATP. In particular, the RMSD values for the holo+ATP species range from 2.0 to 2.5 Å for α2β1 and from 2.7 to 3.0 Å for α2β2 (Table 1). These values are lower than the RMSD values obtained for the apo species (α2β1: 2.5–2.7 Å; α2β2: 2.9–3.4 Å).

**TABLE 1:** RMSD and standard deviation (Å) determined for the protein backbone of the snapshots sampled along the last 500 ns of MD simulations performed for all systems (apo, holo and holo+ATP states) of AMPK isoforms α2β1 and α2β2. Values were determined using the energy-minimized holo+ATP species averaged for the last 200 ns of each simulation system as reference structure.

System		Replica 1	Replica 2	Replica 3	Average
α2β1	Apo	2.6 ± 0.6	2.7 ± 0.4	2.5 ± 0.5	2.6
	Holo	2.5 ± 0.3	2.6 ± 0.5	2.9 ± 0.8	2.6
	holo+ATP	2.5 ± 0.3	2.0 ± 0.2	2.4 ± 0.4	2.3
α2β2	Apo	3.2 ± 0.3	2.9 ± 0.2	3.4 ± 0.5	3.2
	Holo	4.1 ± 0.6	2.7 ± 0.4	3.1 ± 0.3	3.3
	holo+ATP	2.7 ± 0.4	3.0 ± 0.5	3.0 ± 0.4	2.9

Binding of PF-739 to the α2β1 species has no significant effect on the RMSD of the holo species (from 2.5 to 2.9 Å), which is close to the values obtained for the apo form. Only the presence of both the ligand and ATP (holo+ATP) gives rise to a reduction in the RMSD. This effect is even more remarkable in the α2β2 species, as the RMSD of the protein backbone is generally larger than the RMSD value determined for the α2β1 complex in all the states (apo, holo and holo+ATP; see Figure 2 and Table 2). These findings suggest that PF-739 exerts a weak structural stabilization upon binding to both α2β1 and α2β2 species.

**TABLE 2:** Contribution of the essential motion (%) to the structural variance of different AMPK systems and the total contribution of the first four projections.

Systems		Proj. 1	Proj. 2	Proj. 3	Proj.4	Total_(P1-P4)_
α2β1	apo	41.2	12.0	8.1	4.6	66.0
	holo	38.6	12.1	7.6	4.0	62.3
	holo+ATP	30.7	12.6	7.0	5.1	55.4
α2β2	apo	30.9	12.6	8.8	4.8	57.1
	holo	33.1	12.7	8.6	5.3	59.7
	holo+ATP	29.0	13.2	7.2	5.3	54.7

**FIGURE 3:**
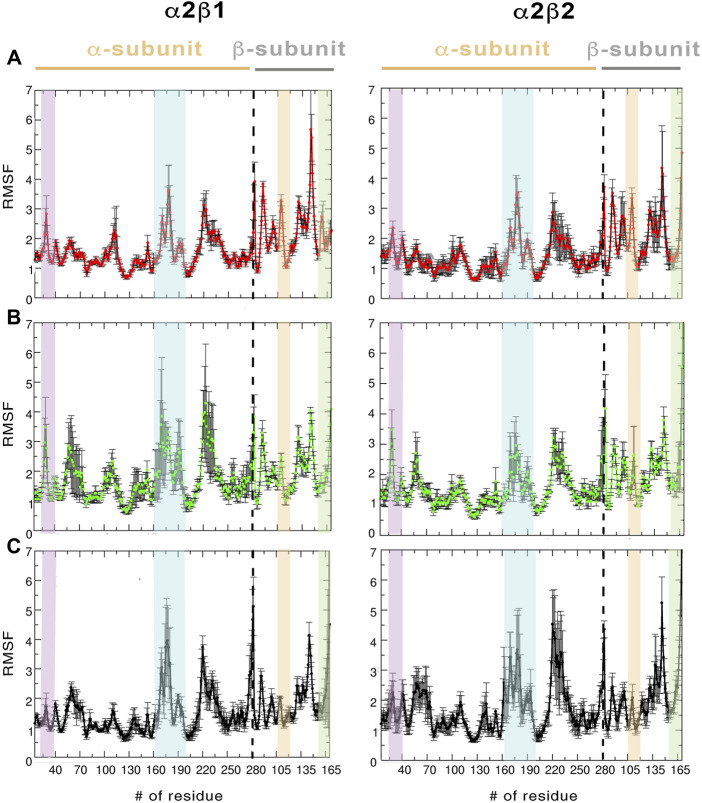
RMSF (Å) average of the residues determined for the protein backbone along the last 500 ns of the three independent replicas runs for **(A)** apo, **(B)** holo and **(C)** holo+ATP. The standard deviation for each residue is shown as an error bar. The highlighted regions denote the moieties corresponding to P-loop **(purple)**, activation loop **(cyan)**, CBM domain **(orange)**, and C-interacting helix **(green)**.

Regarding the per-residue mean square fluctuation (RMSF) profile, similar results are observed for both α2β1 and α2β2 species, as noted in the resemblance of the fluctuation patterns obtained by averaging the RMSF of the three replicas run for every system (Figure 3). The highest fluctuations in the α-subunit correspond to residues in the activation loop (residues 165–185, highlighted in blue in Figure 3) and the α-helix formed by residues 210–230. It is worth noting the higher fluctuation of the P-loop (residues 15–35; purple in Figure 3) in the holo state in comparison to both apo and holo+ATP systems. Thus, binding of PF-739 significantly affects the flexibility of the P-loop in both α2β1 and α2β2 species, which may have functional relevance since the P-loop contributes to shape both the ADaM and ATP-binding sites. Regarding the β-subunit, the largest fluctuations are in the CBM domain, which contains Ser108 (highlighted in orange in Figure 3; phosphorylated in both holo and holo+ATP states), and the regions near the C-interacting helix (residues 162–172, highlighted in green in Figure 3). It is worth noting that the binding of PF-739 (holo) and ATP (holo+ATP) increases the fluctuations of the α-subunit elements mentioned above, while reduces the fluctuations in the β-subunit, independently of the β-isoform. These findings are in agreement with the higher RMSD fluctuations observed in some replicas of the holo states for both α2β1 and α2β2 species.

### Dynamic Properties of AMPK Complexes

In order to examine the effect of the activator on the conformational behavior of AMPK complexes, we have analyzed both the essential dynamics (ED) of the protein backbone and the dynamic correlation between residues.

**FIGURE 4:**
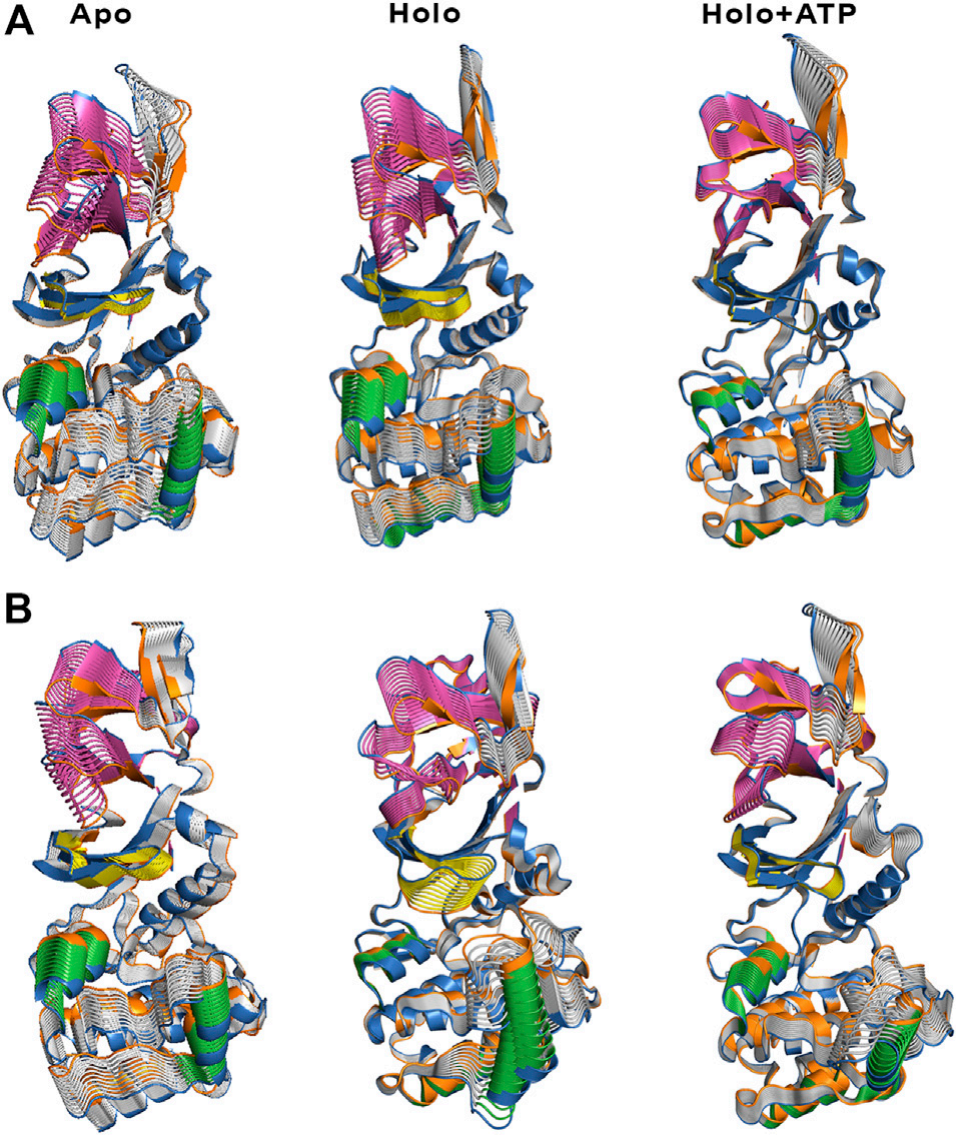
Representation of the first essential motion derived from the ED analysis of the protein backbone for the **(A)** α2β1 and **(B)** α2β2 species, determined from the snapshots sampled along the last 500 ns of MD simulations. The P-loop is shown in yellow, and the helices formed by residues 100–110 and 220–229 in the α-subunit are shown in **green**. The CBM domain is colored in **magenta**.

The ED provides information about the essential motions of the protein and can be used to examine the effect of activator on the major motions of the protein skeleton. The results for the first essential motion for the apo (α2β1 and α2β2) states show a concerted bending that brings α- and β-subunits closer and then moves them apart (Figure 4). The most interesting feature is that the P-loop seems to act as a hinge, assisting the concerted bending between the subunits. Indeed, the first motion accounts on average for 41/31% of the structural variance in α2β1/α2β2 species, and the contribution of the first four motions accounts for 66/57% of the total structural variance (Table 2). This emphasizes the importance of the first essential motion to the conformational flexibility of the AMPK complexes.

Comparison of the ED results obtained for apo, holo and holo+ATP states reveals that binding of the activator has a mild effect on the conformational variance, which is reduced from 66% (apo) to 62% (holo) and 55% (holo+ATP) for the α2β1 species (Table 2). However, for the α2β2 species the activator triggers a slight increase in the conformational variance relative to the apo species, while subsequent binding of ATP results in a reduction of the structural variance (apo: 57.1%; holo: 59.7%; holo+ATP: 54.7%). These results are also reflected in the contribution of the first essential motion (Figure 4 and Table 2). In the holo (α2β1 and α2β2) states, this motion reflects a synchronous motion of the P-loop and the CBM domain, which is in contrast with the increased stiffness observed in the holo+ATP state, especially regarding the P-loop, the helical domain in the α-subunit, as well as the region of the CBM domain nearest to the ADaM site. However, although the movements of the CBM domain are very similar between α2β1 and α2β2 species, the P-loop and the helices at the C-terminal region of the α-subunit exhibit higher fluctuations in α2β2 with respect to α2β1 (Supplementary Figure S1). Finally, it is worth noting that the enhanced stiffness achieved upon ATP binding to holo is again more remarkable in the case of the α2β1 complex (Figure 4 and Table 2).

Besides the qualitative inspection of the overall dynamics of the systems shown in Figure 4, we have determined the similarity indices for the first essential motions (Supplementary Table S1). The similarity index for the apo species (i.e., the most flexible one) is close to 0.70 and 0.60 for α2β1 and α2β2, respectively, reflecting the preservation of the major deformation of the protein skeleton in the three replicas. These results also agree with the higher conformational flexibility observed for α2β2 systems. In the holo species, the similarity indices are 0.75 for α2β1 and 0.47 for α2β2 systems. These results agree with the essential motion observed for the holo state of α2β1 (Figure 4A, middle panel) and α2β2 (Figure 4B, middle panel). In the former, the variance of the system is more balanced between certain regions, i.e., CBM domain, P-loop, A-loop and helices P220-G229 and E100-R110 (colored in green, Figure 4). However, higher fluctuations account for the structural elements in the α-subunit in α2β2. These findings are in agreement with the previous RMSD and RMSF results. Finally, for the holo+ATP systems the similarity index is close to 0.35 for α2β1 and α2β2, respectively. However, this simply means that binding of both activator and ATP rigidifies the protein skeleton, annihilating the large-scale deformations observed in the apo species as observed in Figure 4. The ED, shown in Figure 4, as well as the similarity indexes calculated, in Supplementary Table S1, have been obtained considering the last 500 ns of the simulation time of the three replicas. However, in order to check the statistical value of our simulations, we have also calculated the similarity indexes for the first three essential motions of the apo α2β1 and α2β2 derived from the ED analysis in time windows 200–600 and 600–1,000 ns for the three replicas (Supplementary Table S2). The similarity index amounts in general to 0.8. For the first replica of α2β1 system a lower similarity is observed, suggesting a slower structural relaxation, as noted in the similarity obtained for more advanced time windows (Supplementary Table S3). Overall, these results suggest that selection of the last 500 ns to perform the statistical analysis of the simulations is well suited for the comparison between replicas, although these results also suggest that shorter time periods might be also usable. For this reason this 500 ns time window has been used in further analysis.

**FIGURE 5:**
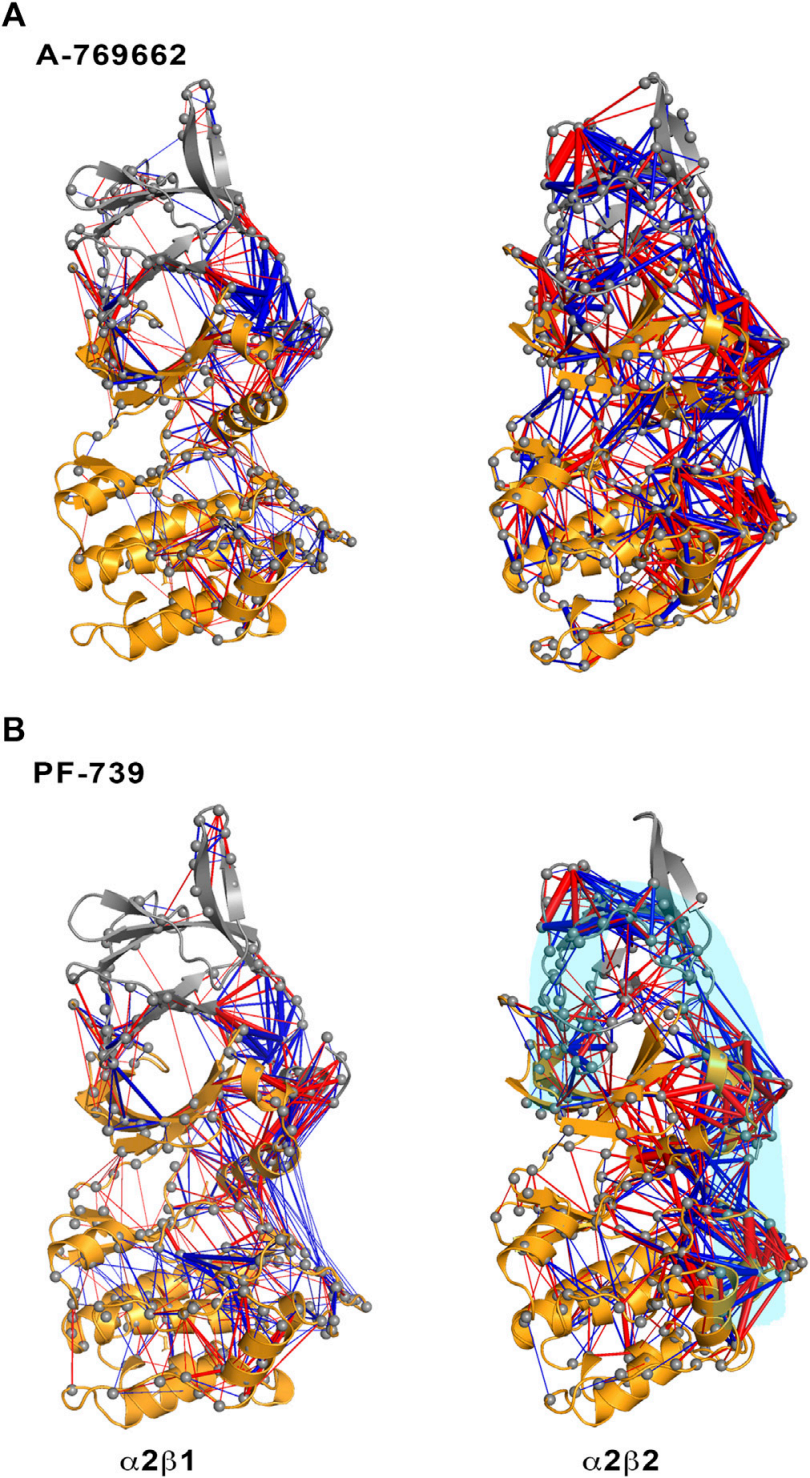
Representation of the contact changes observed in the dynamical perturbation network between apo and holo states for **(left)** α2β1 and **(right)** α2β2 species. The holo states were calculated in presence of **(A)** A-769662 and **(B)** PF-739 activators. Red/blue edges show interresidue atomic contact increase/decrease in the holo state relative to the apo form. The magnitude of these changes is indicated by the width of the edges.

**FIGURE 6:**
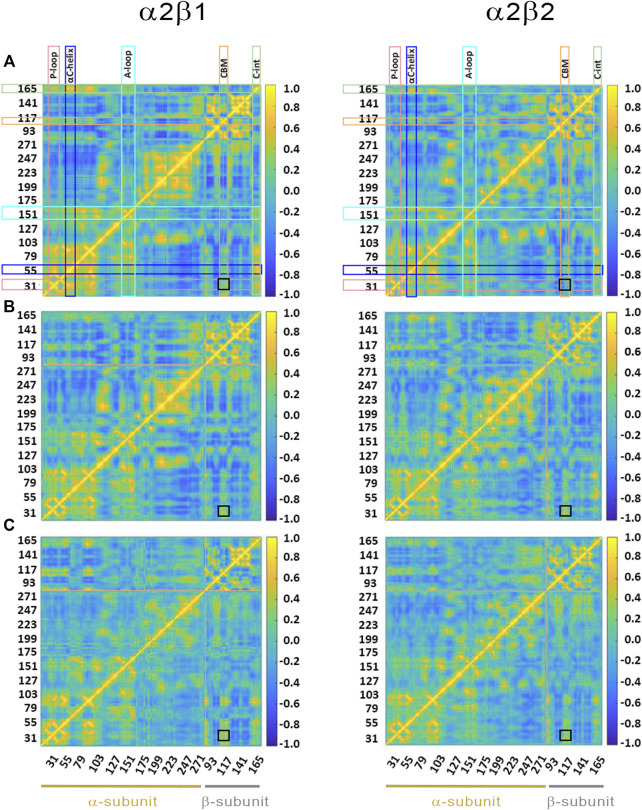
Dynamic cross-correlation (DCC) matrices for **(A)** apo, **(B)** holo and **(C)** holo+ATP complexes of **(left)** α2β1 and **(right)** α2β2 with PF-739. The *x*- and *y*-axis denote the numbering of residue in the α- and β-subunit, indicated at the bottom of the plot (yellow and gray lines for α- and β- subunits, respectively). Specific regions of AMPK are highlighted with squares at the top of the plot: P-loop **(pink)**, αC-helix **(blue)**, activation loop (A-loop; **cyan**), CBM unit **(orange)** and C-interacting helix (C-int; **green**). Regions colored in yellow/blue show high correlated/anticorrelated fluctuations. The black square in the plots highlights the motion between the CBM domain and the P-loop.

To complement the results of ED analysis, we have performed two additional analyses with the aim to assess the dynamic correlation between residues and disclose specific relationships between the α- and β-subunits: a dynamical perturbation network (DPN, Figure 5) and a dynamic cross-correlation (DCC, Figure 6; see *Methods and Materials* for technical details) analysis.

The dynamical perturbation network (DPN) was calculated for apo and holo species as an average of the three independent replicas. Figure 5 shows the changes in the correlation of residues between apo and holo states, where blue/red edges stand for contacts weakened/strengthened in holo relative to apo state. Thus, these networks provide information of how the interaction of the activator with the enzyme affects the contact network between residues. For the sake of comparison, this analysis was performed not only for PF-739, but also for A-769662, which exhibits a marked selectivity for β1-containing AMPK complexes. Our previous studies (Human Protein Atlas (2021, 2021; Ngoei et al., 2018) revealed that A-769662 acts as molecular glue between the α2- and β1-subunits, while this effect is lost in the α2β2 species due to the higher dynamical resilience of this specie towards the activator. The dynamical contact network for A-769662 (Figure 5A) perfectly agrees with these findings. In fact, the changes between apo and holo in α2β1 mainly reveal a higher number of contacts between the P-loop of the α2-subunit and the CBM of the β1-subunit as well as between the αC-helix of the α2-subunit and the C-interacting helix of the β1-subunit. Conversely, the contact network that emerges for the α2β2 complex is more complex, involving regions located far from the ADaM site. This result agrees with the higher flexibility of the α2β2 species, and the lower impact of A-769662 on the dynamical response of this complex.

For the pan-activator PF-739, the α2β1 complex exhibits fewer and more specific contacts, which primarily affect the CBM/P-loop and the αC-helix/C-interacting helix/A-loop, than the α2β2 species, thus resembling the results discussed for A-769662. However, the number of contacts weakened or even lost between the A-loop and the αC-helix in the α2β1 holo state is remarkably higher for PF-739-bound complexes compared to A-769662-bound ones (Figures 5A,B, left side). For α2β2, the number and weights of the edges are larger in this species, and the distribution of contacts involves wider regions from the CBM domain to the A-loop (see highlighted region in cyan, Figure 5B, right panel). Noteworthy, DPN analysis reveals that binding of A-769662 gives rise to a much larger difference in the dynamical network of α2β1/A-769662 and α2β2/A-769662 complexes than for α2β1/PF-739 and α2β2/PF-739 complexes, as the pattern observed for the last two AMPK complexes exhibit a similar pattern (Figure 5). This is in agreement with the experimental results that indicate that the PF-739 is active against both β1- and β2-containing isoforms, in contrast with the selective activation of β1-containing AMPK complexes reported for A-769662.

Finally, the dynamic cross-correlation (DCC) analysis was performed to examine the correlated motions of residues in α2β1 and α2β2 AMPK complexes. For the apo systems (Figure 6A) one may notice a significant correlation between residues in the P-loop and the αC-helix, both in the α-subunit, and between the αC-helix from the α-subunit and the C-interacting helix from the β-subunit (as noted by the yellow marks). It is worth noting that there is a slight correlation between the P-loop and the CBM domain (β-subunit), more remarkable in α2β1 than in α2β2, as noted by the similarity indexes of 0.82 for α2β1, which is reduced to 0.75 in α2β2 (Supplementary Table S4). The holo+ATP systems show lower dynamical correlation between residues, as observed by the progressive reduction in the number and intensity of the areas that exhibit a pronounced correlation (shown in yellow and blue for highly correlated and anticorrelated fluctuations between residues, respectively). On the contrary, the correlation between the motion of the P-loop and the CBM domain is reinforced in the holo and holo+ATP states (black square in Figure 6). These effects are more noticeable for the comparison of holo in α2β1 (similarity indices of 0.63 in α2β1 vs 0.55 in α2β2, Supplementary Table S4), while lower differences exist for holo+ATP systems in α2β1 and α2β2, in agreement with previous analyses.

Although the preceding results show a high similarity in the dynamical behavior of both α2β1 and α2β2 species bound to PF-739 activator, which agrees with the definition of PF-739 as a pan-activator, these analyses still reveal subtle differences between β1- and β2-containing AMPK complexes. In particular, the results suggest that the α2β2 species have a larger resilience to the structural modulation exerted by the activator, whereas the α2β1 isoform is more sensitive to the conformational adaptation induced upon activator binding to the ADaM site, enhancing the stiffness of protein backbone for the β1-containing complex (Figures 4, 5). These results agree with the fact that PF-739, which can activate both α2β1γ1 and α2β2γ1 complexes, still exhibits a larger affinity for the β1-isoform (Figure 1) (Cokorinos et al., 2017).

### Pre-Organization of ATP-Binding Site

To explore how PF-739 could influence the activation of AMPK, we have evaluated the dynamical response of the ATP-binding site due to the binding of the activator in the ADaM site. Specifically, we have assessed the pre-organization of the ATP-binding site in the apo, holo and holo+ATP states, using as a reference the average structure of the holo+ATP complex.

**FIGURE 7:**
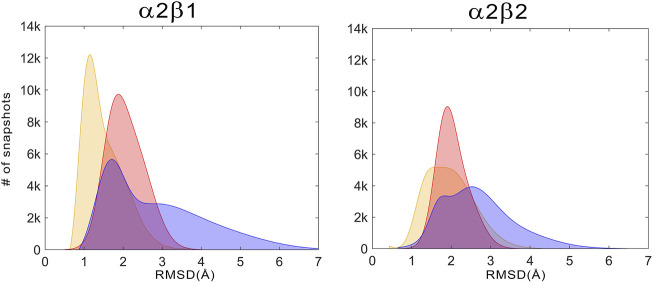
Distribution of the positional deviation (RMSD; Å) of the structures sampled along the trajectories run for apo **(red)**, holo **(blue)**, and holo+ATP **(yellow)** for the residues that shape the ATP-binding site (residues α22–α32, α42–α46, α75–α79, α142–α147, and α153–α157). A total of 60,000 snapshots taken from the last 500 ns of MD simulations were considered for each system in the analysis.

For the holo+ATP states, the residues of the ATP-binding site sample a conformational space with a high peak centered at a positional RMSD of 1.2 Å and a shoulder at 1.9 Å for α2β1, while a wider distribution is observed with a peak centered at 1.8 Å for α2β2 (Figure 7, Gaussian distributions colored in yellow). Unexpectedly, the apo state shows a narrower distribution with a unique peak centered at 2.0 Å for both α2β1 and α2β2 species. In fact, the conformations sampled by the apo state have a notable overlap with the distribution of holo+ATP, this resemblance being more significant for the α2β2 species. In contrast, the holo state exhibits a wider distribution, showing a bimodal RMSD profile, with peak values at 1.7 and 3.2 Å for α2β1, and at 1.8 and 2.5 Å for α2β2. These results suggest that the binding of PF-739 enhances the fluctuations of P-loop residues that shape the ATP-binding. Due to this higher conformational flexibility, the ATP-binding site can adopt conformations close to those populated in the holo+ATP state, but also visit more dissimilar conformational regions even in comparison with the apo state (Figure 7).

### Structural Basis of the AMPK Activation by Pan-Activator PF-739 and Its Comparison With Other Direct Activators

**FIGURE 8:**
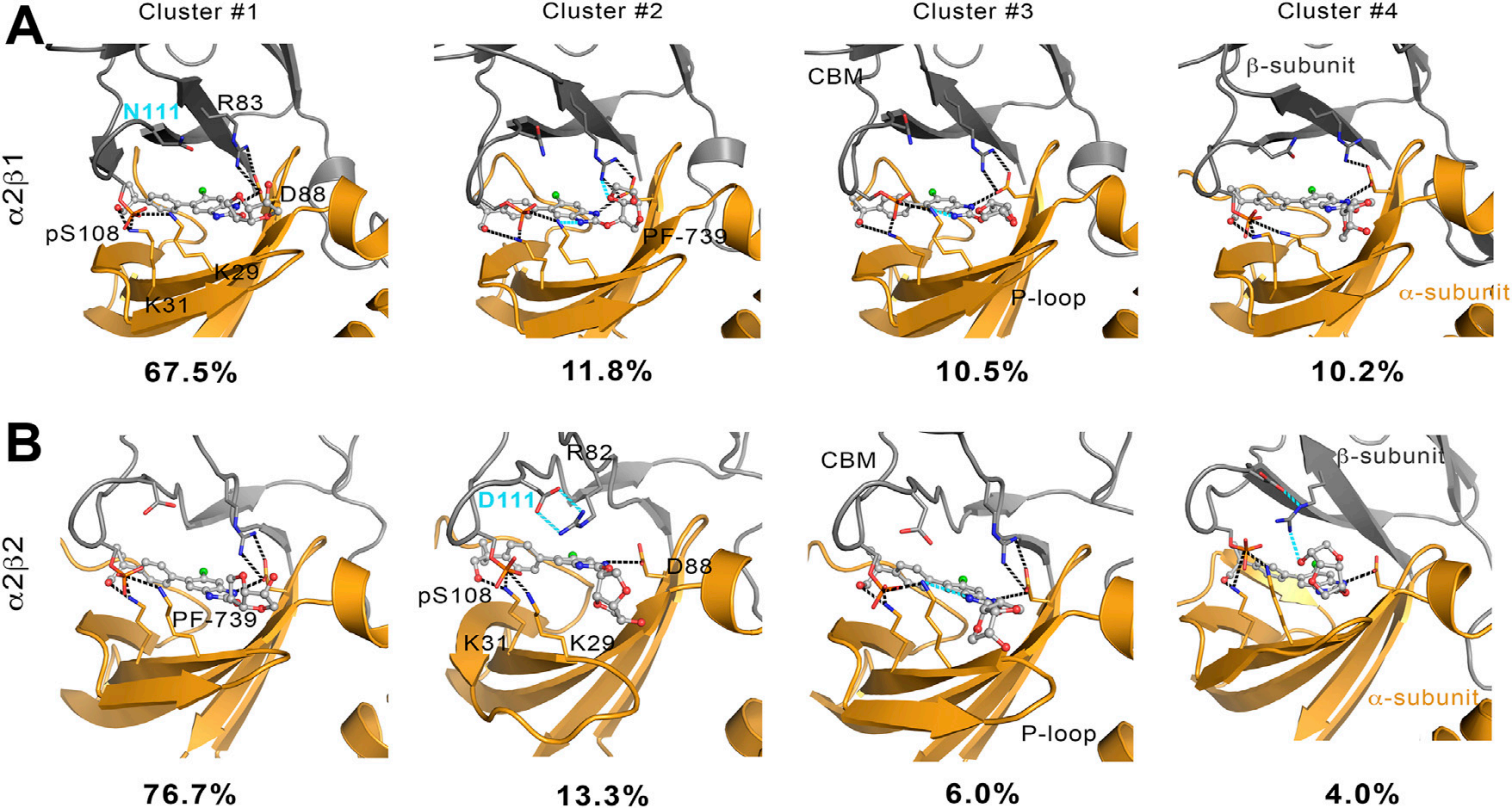
Representation of main interactions between the CBM, P-loop and PF-739 for holo states of **(A)** α2β1 and **(B)** α2β2 species for the four clusters obtained along the last 500 ns of simulation of each replica. The α-subunit is shown in orange cartoon, while the β-subunit is shown in grey cartoons. PF-739 is shown in grey ball and sticks in the ADaM site. Selected polar interactions maintained through all the MD simulations and clusters are highlighted in black dashed lines, while those formed in specific clusters are shown in cyan.

To complement the previous analyses, we have examined the interaction network formed by PF-739 and the residues in both α- and β-subunits. To this end, we have clustered the snapshots sampled along the last 500 ns simulation of each replica for both holo α2β1 and α2β2 species, summing a total of 1.5 μs. The results for holo-α2β1 system display up to 4 different clusters, which account for 67.5, 11.8, 10.5 and 10.2% of the conformational ensemble, where the main difference is the conformation adopted by the sugar-like mannitol ring appendage of PF-739 (Figure 8A). In all cases two regions can be identified in the interaction network. The first one corresponds to the salt bridge formed between β1-Arg83 and α2-Asp88 (3.0 ± 0.3 Å), which at the same time is hydrogen-bonded to PF-739 (3.5 ± 0.6 Å). For the second cluster (11.8%), an additional interaction between β1-Arg83 and the sugar-like mannitol ring is observed (3.7 ± 0.6 Å; Figure 8A). The second region involves salt bridges between pSer108 located at the β-subunit CBM domain and α2-Lys29 (3.7 ± 0.9 Å) and α2-Lys31 (4.4 ± 1.3 Å), both from the P-loop of the α-subunit. Moreover, α2-Lys29 and α2-Lys31 establish contacts with PF-739, such as a hydrogen bond between the Lys31 and the hydroxymethyl-cyclopropyl group (3.2 ± 0.7 Å), which is found in all clusters, and an additional interaction between Lys29 and the N of the benzimidazole ring (3.9 ± 0.9 Å, Figure 8A) present in clusters 2 and 3. These interactions networks are very similar to those found in our previous study of SC4 (Aledavood et al., 2021), suggesting that the structural differences between these two compounds, mainly regarding the *o*-toluic substitution of SC4 by mannitol-like ring appendange in PF-739, and the 4′-nitrogen of imidazopyridine in SC4 by a carbon atom in PF-739, do not have a dramatic effect over the interaction at the ADaM site (see also Supplementary Table S5). Indeed, these findings remark the key role of the β1-Arg83/β2-Arg82 in the organization of these interactions networks as we explain below.

**FIGURE 9:**
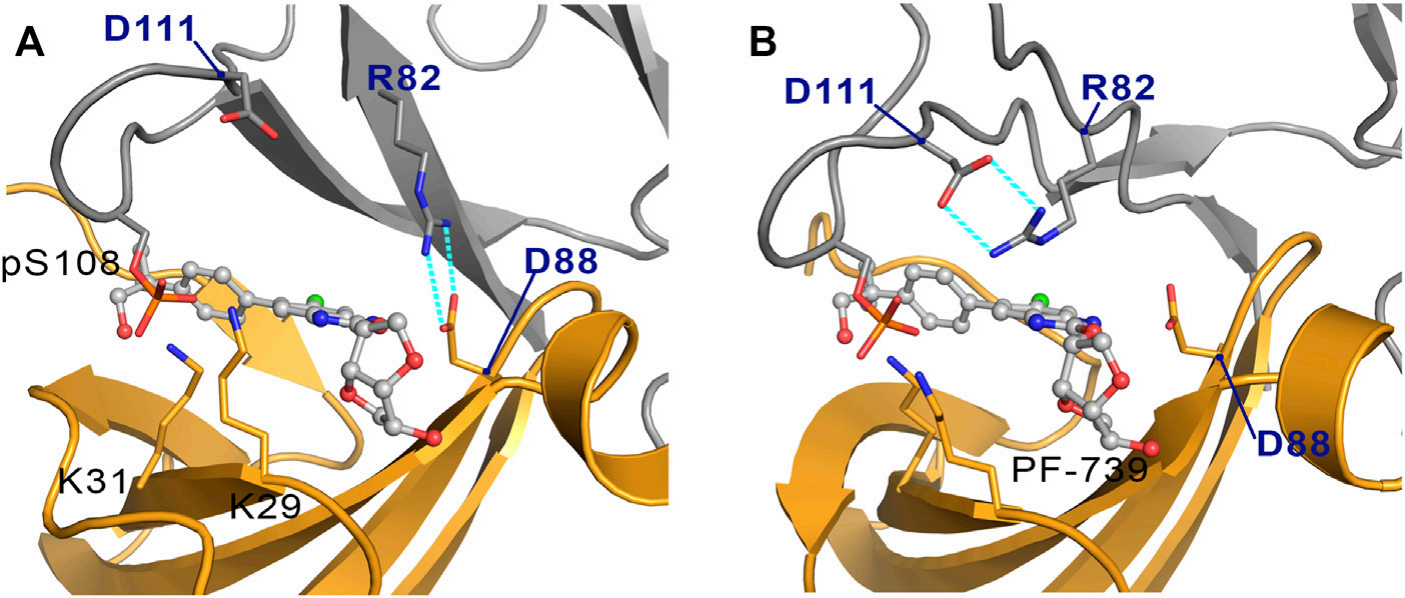
Representation of the two orientations of the β2-Arg82 in α2β2 species, where the interaction with **(A)** the α2-Asp88 and **(B)** the β2-Asp111 are highlighted in cyan dashed lines. The α-subunit is shown in orange cartoon, while the β-subunit is shown in grey cartoons. PF-739 is shown in grey ball and sticks in the ADaM site.

The cluster analysis performed for the holo-α2β2 system yields four clusters that differ in the orientation of the sugar-like mannitol ring of PF-739, accounting for 76.7, 13.3, 6.0 and 4.0% of the structural ensemble (Figure 8B). However, these clusters show higher structural diversity than those determined for the holo-α2β1 system. Thus, two distinct orientations of β2-Arg82 are found in all clusters (Figure 9). In one case (Figure 9A), β2-Arg82 interacts with α2-Asp88 (3.9 ± 1.3 Å), which forms a hydrogen bond with PF-739 (2.9 ± 0.2 Å). This arrangement represents 54.4% of all the conformations sampled for the α2β2 holo species. In the second orientation β2-Arg82 interacts with β2-Asp111 (3.8 ± 0.9 Å), accounting for 45.6% of the conformational ensemble (Figure 9B). Notably, in the α2β1 holo species this latter interaction is not observed, which can be attributed to the substitution of β2-Asp111 by β1-Asn111. The second orientation found for β2-Arg82 reinforces the interaction network observed through β-pSer108, which maintains its interactions with both αLys29 (3.3 ± 0.6) and αLys31 (3.8 ± 1.0) from the P-loop. Additionally, the interaction between αLys31 and the hydroxymethyl-cyclopropyl group (3.2 ± 0.6 Å) of PF-739 is maintained in all clusters, while the interaction between Lys29 and the N of the benzimidazole ring is less stable and only slightly observed in cluster #3 (4.4 ± 0.7 Å, Figure 8B).

These results suggest that the arrangement of the sugar-like mannitol unit structural, which exhibit notable differences between clusters, does not have a significant impact on the interaction network observed along the simulations, since the main interactions are preserved in all cases. Indeed, the arrangement of the sugar-like mannitol ring gives rise to new interactions between β1-Arg83 (β2-Arg82) and PF-739 only in cluster #2 (11.8%) for α2β1 and cluster #4 (4.0%) for α2β2. Furthermore, the conformation of the β1-Arg83/β2-Arg82 residue emerges as a key structural feature. While in the α2β1 holo specie, β1-Arg83 forms a salt bridge with α2-Asp88 in all sampled conformations, two orientations are found for β2-Arg82 in the α2β2 holo species (Figure 9). This distinctive trait can be attributed to the substitution β1-Asn111 → β2-Asp111, since the presence of β2-Asp111 in α2β2 promotes an electrostatic competition with α2-Asp88 for the interaction with β2-Arg82.

**FIGURE 10:**
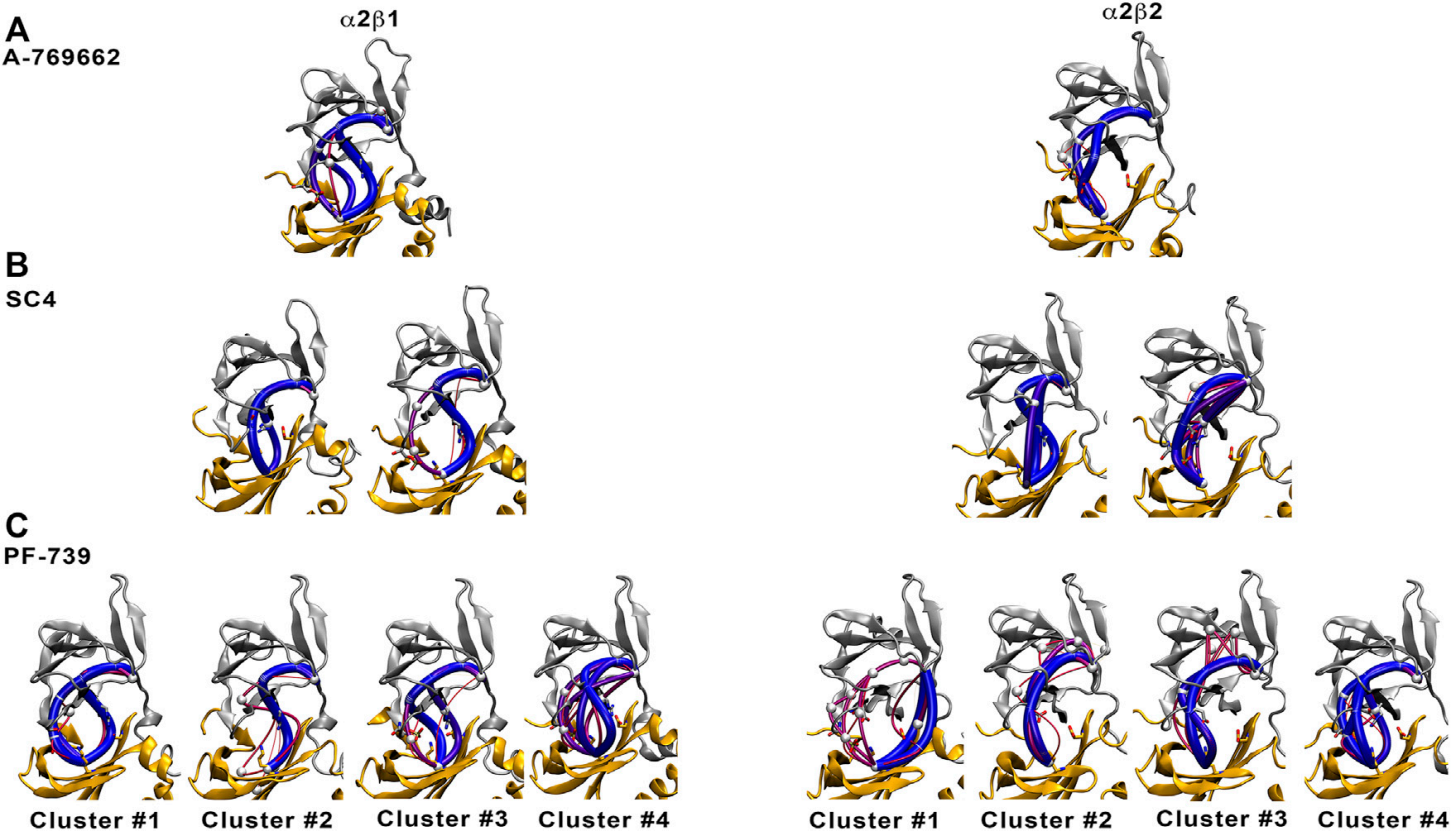
Comparison of major interaction networks obtained from WISP analysis for α2β1 **(left panel)** and α2β2 **(right panel)** species of the holo states for **(A)** A-769662, **(B)** SC4 and **(C)** PF-739 direct activators.

To confirm these results, we have calculated the major interaction pathways identified from WISP analysis for the holo species formed with PF-739. Figure 10 show the WISP results obtained in our previous work (Ngoei et al., 2018) for A-769662 (Figure 10A) and SC4 (Figure 10B), as well as the results obtained for PF-739 (Figure 10C). For the α2β1/A-769662 complex three major paths are found between the CBM domain and the P-loop, which involve i) pSer108, ii) the hydrophobic core of the ADaM site, and iii) the interaction β1-Arg83-α2-Asp88. All of them are directly connected with the activator through the residues participating in the path, supporting the role of A-769662 as a molecular glue between α2- and β1-subunits. However, only the pSer108 path is observed for the α2β2/A-769662 complex. This can be attributed to the β1-Asn111 → β2-Asp111 substitution, weakens the interaction between β2-Arg82 and α2-Asp88, and strengthens the path through pSer108. In turn, this agrees with the selective activation observed for AMPK complexes containing the β1-isoform. In contrast, two representative paths are found in the holo states formed with SC4 (Figure 10B), corresponding to the networks through pSer108 and through the pair β1/2-Arg83−α2-Asp88. Furthermore, SC4 exhibit a similar pattern in both α2β1 and α2β2, which is in agreement with the ability to activate both kinds of AMPK complexes (Hardie, 2014). Interestingly, the β1-Asn111 → β2-Asp111 substitution seems to be less sensitive to the presence of SC4, an effect that can be attributed to the negative charge of the activator that can modulate the linking role of β2-Arg82 towards a preferential interaction with either β2-Asp111 and α2-Asp88.

In light of these findings, we have performed the WISP analysis separately for the four main clusters obtained for PF-739 (Figure 10C). In the case of the holo-α2β1 state, the three pathways described above for A-769662 can be identified in the whole set of clusters. Although one may notice distinct traits for each cluster, at least two main paths can be observed for clusters #1, #3 and #4. In particular, for the most populated cluster (#1; 67.5%) they correspond to the paths mediated by pSer108 and the pair β1-Arg83−α2-Asp88, respectively. However, the analysis of the holo-α2β2 state reveals a weaker connectivity, since a single path dominates the interaction network in all clusters, For the most populated cluster #1 (76.7%), the path involves the β2-Arg82−α2-Asp88 pair, with a minor contribution of the pSer108-mediated path. In the other clusters, nevertheless, the pSer108 path is predominant, resembling the behavior found for A-769662 (Figure 10A, right panel).

These results suggest that the β1-Asn111 → β2-Asp111 substitution plays a critical role in defining the mechanical sensitivity of AMPK to the direct activator. Besides the pSer108-mediated path, the presence of β1-Asn111 in α2β1 favors the formation of an additional path that involves the concerted interaction between β1-Arg83, α2-Asp88, activator and α2-Lys29/α2-Lys31. Nevertheless, the substitution β1-Asn111 → β2-Asp111 favors the breaking of the β2-Arg82−α2-Asp88 interaction and the formation of the salt bridge with β2-Asp111, which reinforces the contribution of the pSer108 path, making the α2β2 complex less sensitive to the modulation by the activator.

**FIGURE 11:**
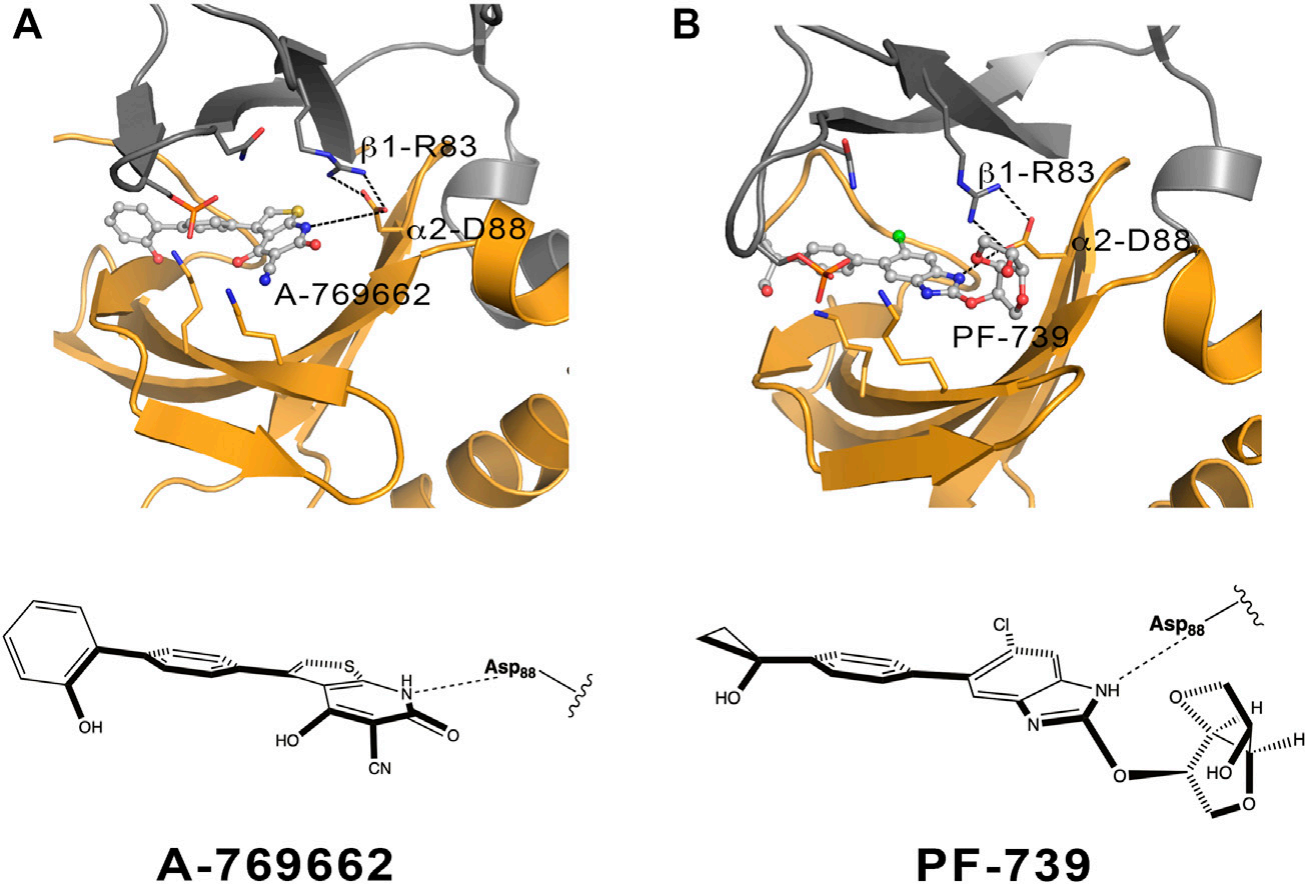
Schematic representation of the α2β1 holo state for **(A)** A-769662 and **(B)** PF-739. The 2D chemical structure of the direct activators is presented with the orientation displayed in the ADaM site. The polar interactions between β1-Arg83, α2-Asp88 and the respective ligands are shown in dashed lines.

The chemical features of the activator also exerts role in assisting the conformational activation of both α2β1 and α2β2 species. The main difference between A-769662 and PF-739 is the replacement of the thienopyridone ring by a benzimidazole derivative with a sugar-like mannitol appendage in PF-739 (Figure 11). The β1-Arg83−α2-Asp88−A-769662−α2-Lys29/α2-Lys31 network of interactions acts as a transmission band that connect the dynamical motion of the CBM domain with the P-loop, assisting the effective transition toward conformations that resemble the ATP-binding site in the holo+ATP state for the α2β1 species (Supplementary Figure S2, left). However, breakage of this interaction path in the α2β2 holo species prevents the activator to mediate the transmission of the dynamical fluctuations of the CBM domain and the P-loop, which is reflected in a wider conformational distribution of the ATP-binding site (peak centered at 3.0 Å; see Supplementary Figure S2, top). This reflects the inability of A-769662 to pre-organize the ATP-binding site in β2-containing AMPK complexes.

The conformational response caused by PF-739 is more complex, reflecting the structural variability of the clusters regarding the orientation of the sugar-like mannitol appendage for both α2β1 and α2β2 species, and the two arrangements of β2-Arg82 in α2β2 compared to the single conformation of β1-Arg83 in α2β1. The analysis of the pre-organization of ATP-binding site (Figure 7) reveals that the activator is unable to reduce the conformational sampling to structures well suited for the binding of ATP, which would diminish the activation effect of PF-739. At this point let us remark the bimodal behavior shown in Figure 7A, with only 33.3/45.0% of the sampled structures of ATP-binding site resembling the holo+ATP in α2β1/α2β2, whereas A-769662 triggers a marked shift in the population distribution in the holo complex of α2β1 (Supplementary Figure S2). On the one side, this agrees with the ability of PF-739 to exert a mild activation in both α2β1 and α2β2. The distribution of holo+ATP−*like* conformations in α2β2 is wider than in α2β1, which reflects the higher structural plasticity observed in α2β2 species. On the other side, these findings are also in agreement with the WISP results, which show how PF-739 activator has higher gluing effect than A-769662 in α2β2, allowing the transmission of the information between α- and β-subunit through the pSer108 and β2-Arg82−α2-Asp88 pathways, explaining in this way why PF-739 acts as a pan-activator.

## Conclusion

Discerning the molecular factors that regulates the structure-function relationships of AMPK isoforms is of utmost importance to rationalize the tissue-dependent expression of AMPK complexes, and thus enabling the design of specific compounds active against specific metabolic disorders. However, the recognition of the differences between isoforms that allow a different ligand behavior (i.e., selective activator, pan-activator or even inhibitor) is very challenging due to the high structural complexity of the enzyme and the highly correlated dynamics observed for both α2β1 and α2β2 species.

Our results confirmed that the subtle difference of β1-Asn111 to β2-Asp111 has great implications in the dynamical response of AMPK to the binding of activators. This single substitution can change the interaction networks formed surrounded the activator, thus inducing a better mechanical response of the α2β1 specie towards the interaction of PF-739, than in the case of the α2β2 species. So, even in case of a pan-activator like the PF-739, able to activate both β-isoforms, still subtle residue substitutions in the ADaM site are responsible of difference in affinity towards the isoform. Additionally, we hypothesized that the bulkier substitutions in the chemical structure of the ligands located nearest to the α2-Asp88 residue could involve a higher variability in the conformational space, thus preventing to discern between β-isoforms.

In summary, we were able to characterize the key molecular features that mediate the activation of pan-activator towards α2β1and α2β2 species. All these findings shed light in the comprehension of the role of specific residues in the ADaM site that can modulate or completely change the direct activation mechanism of β1- and β2-containing AMPK complexes. Future studies will be appreciated to distinguish the structural basis of the different sensitivity of AMPK complexes formed by distinct α-subunits, and which is more important, the study of the full complex to disentangle the full allosteric network connection. This understanding will really enable us the design of tissue-selective modulators of this cellular energy sensor.

## Materials and Methods

### Molecular Dynamics Simulations

Extended molecular dynamics (MD) simulations were utilized to analyze the structural and dynamical characteristics of the simulated system. For this purpose, the α2β1γ1 systems were built up using the complexes with A-769662 (PDB entry 4CFF) (Xiao et al., 2013). On the other hand, the system related to the complex of α2β2γ1 bound to SC4 (PDB entry 6B2E) (Ngoei et al., 2018) was also used as a template to model the complexes with PF-739. Following our previous studies, (Aledavood et al., 2019; Aledavood et al., 2021), the γ-subunit was not considered in MD simulations for several reasons. First, the ADaM site is shaped only by α- and β- isoforms. Furthermore, the lack of precise structural information about stretches of both α- and β-subunits, particularly regarding the C-terminal regions, which are located close to the γ-subunit, would introduce an additional level of uncertainty, opening the way to potential artefacts in the simulations. Finally, inclusion of the γ-subunit would have required a larger computational cost to guarantee a proper sampling of the dynamical motions of the three isoforms. Accordingly, following the “divide-and-conquer” strategy outlined above, the simulated systems comprise only α- and β-subunits. Specifically, simulations were performed for residues 8–278 of the α2 isoform, and residues 78–173 and 77–171 of the β1- and β2-isoforms, which were solved without disruptions in the X-ray structures. Finally, these structures were used to model the apo protein, the complexes of the activators bound to the phosphorylated Ser108 (pSer108)-containing isoforms (holo), and the corresponding holo+ATP complexes with both activator in the ADaM site and ATP in the ATP-binding site.

The Molecular dynamic (MD) simulations were performed using the AMBER18 package (Case et al., 2018) and the Amber ff99SBILDN force field (Lindorff-Larsen et al., 2010) for the protein, whereas the ligand (PF-739) were parameterized using the GAFF force field (Wang et al., 2004) in conjunction with restrained electrostatic potential-fitted (RESP) partial atomic charges derived from B3LYP/6-31G(d) calculations (Bayly et al., 1993). The parameters used for the ATP molecule were obtained from the Amber parameters database from Bryce group at the University of Manchester (AMBER parameter database, 2021; Meagher et al., 2003). The standard protonation state at physiological pH was assigned to ionisable residues, and a capping group (N-methyl) was added to the C-terminus of the α-subunit. The simulated systems were immersed in an octahedral box of TIP3P water molecules considering a solute-edge distance of 12 Å (Jorgensen et al., 1983), and counterions atoms were added to maintain the neutrality of the simulated systems (Joung and Cheatham, 2008). The final systems included the AMPK protein (368 residues for α2β1 and 367 residues for α2β2), around 25,000–26,700 water molecules, and a variable number of Na^+^ and Cl^−^ ions, leading to simulated systems containing between 81,000 and 86,000 atoms (specific values are gathered in Supplementary Table S6).

Simulations were performed in the NPT ensemble for equilibration and NVT for MD productions using periodic boundary conditions and Ewald sums (grid spacing 1 Å) for treating long-range electrostatic interactions. Apo, holo and holo+ATP systems were simulated in triplicate. The minimization of the systems was performed refining the position of hydrogen atoms in the protein (2,000 cycles of steepest descent algorithm followed by 8,000 cycles of conjugate gradient), subsequently minimizing the position of water molecules (using again the previous scheme), and finally minimization of the whole system (4,000 cycles for steepest descent and 1,000 cycles of conjugate gradient). Later, the temperature of the system was gradually raised from 100 to 300 K in five steps, 50 ps each using the NVT ensemble and Langevin dynamics for the temperature regulation. In this process, suitable restraints (5 kcal mol^−1^ Å^−2^) were imposed to keep the ligand (activator, ATP) in the binding pocket and prevent artefactual rearrangements along the equilibration stage. In order to equilibrate the density of the system an additional 5 ns step performed in the NPT ensemble using the Berendsen barostat. In addition, the restraints were progressively eliminated in this later step. Production MD simulations were run for 1 μs per replica, leading to a total simulation time of 12 μs for the ligand-bound AMPK complexes, and 6 μs for the two apo species of AMPK.

### Essential Dynamics

This method was utilized to specify the most important motions from the structural variance sampled in MD simulations. In essential dynamics (ED) (Amadei et al., 1993), the dynamics along the individual modes can be studied and visualized separately, so we can filter the main collective motions during our simulations. Therefore, the positional covariance matrix is created and diagonalized in order to achieve the collective deformation modes, i.e., the eigenvectors, while the eigenvalues account for the contribution of each motion to the structural variance of the protein. ED analysis was done for 25,000 snapshots from the last 500 ns of each simulation, taking into account only the backbone atoms and the calculations were performed with PCAsuite program (available at http://www.mmb.irbbarcelona.org/software/pcasuite/pcasuite.html), which is integrated in the pyPCcazip program, a suite of tools for compression and analysis of molecular simulations (Shkurti et al., 2016).

### Dynamical Perturbation Network

Contact networks represent a protein as a collection of nodes, i.e., the residues that are connected by edges if those residues satisfy a contact condition. Here, in line with previous works (Vuillon and Lesieur, 2015; Dorantes-Gilardi et al., 2018; Gheeraert et al., 2019), the contact condition is satisfied if at least one heavy atom from a residue is at a distance below 5 Å from a heavy atom of another residue. Edges are then weighted by the total number of atomic couples that satisfy this contact condition. Individual contact networks from the frames of one MD simulation are built and averaged (considering the average total number of atomic contacts from various replicas) in order to create a dynamical weighted contact network, which represents a time-averaged contact network associated to the corresponding MD simulations.

To compare MD simulations of a protein in various states (i.e., apo, holo and holo+ATP complexes), we computed perturbation contact networks (Gheeraert et al., 2019) by subtracting two dynamical weighted contact networks associated to each pair of states. To differentiate increases and decreases in contact we assign colors to the edges of the dynamical perturbation network according to the sign of its edges. Finally, for visualization purposes a weight threshold can be applied so that only edges wit a weight greater than the threshold are kept for visualization, here set to 5 as in previous work (Gheeraert et al., 2019). Nodes isolated after this process are also pruned to simplify the visualization.

### Dynamic Cross-Correlation Analysis

To complement the information gained from the ED analysis, dynamic cross-correlation (DCC) was used to examine the correlation motion of residues along a given trajectory. To this end, all the snapshots were aligned by means of least-square fitting of Cα atoms of the whole protein to the equilibrated starting configuration. Then, the DCC matrix was determined as noted in Eq. 1.

$${\text{C}}_{\text{ij}}=\frac{{\text{c}}_{\text{ij}}}{{\text{c}}_{\text{ii}}^{1/2}{\text{c}}_{\text{jj}}^{1/2}}=\frac{\langle {\text{r}}_{\text{i}}{\text{r}}_{\text{j}}\rangle -\langle {\text{r}}_{\text{i}}{\text{r}}_{\text{j}}\rangle }{{\left[\left(\langle {\text{r}}_{\text{i}}^{2}\rangle \langle {\text{r}}_{\text{i}}^{2}\rangle \right)\left(\langle {\text{r}}_{\text{j}}^{2}\rangle -\langle {\text{r}}_{\text{j}}^{2}\rangle \right)\right]}^{1/2}}$$ (1)

where the position vectors of two Cα atoms i and j fitted in the structure at time t are denoted as r_i_(t) and r_j_(t), respectively.

The cross-correlation coefficients range from −1 to +1, which represent anticorrelated and correlated motions, respectively, whereas values close to zero indicate the absence of correlated motions (Hünenberger et al., 1995). This analysis was performed using the module available in AMBER package. The similarity between the DCC matrices computed for the three replicas run for apo, holo and holo+ATP systems was estimated using the Tanimoto similarity index. This parameter is a distance metrics used to quantify the degree of similarity between two sets of data. While this index is widely adopted to compare the descriptors that characterize the chemical structure of molecules, in this study it is used to compare the correlated motions determined for pairs of residues in the AMPK complexes.

### Cluster Analysis

Cluster analysis is a way of determining structure populations from MD simulations. Clustering results in a partitioning data so that data inside a cluster are more similar to each other than they are outside a cluster. In MD, this is a mean of grouping similar conformations together. Similarity is defined by a distance metric, the smaller the distance, the more similar the structures. We used coordinate RMSD as the distance metric parameter. Additionally, we used K-means algorithm as implemented in cpptraj software (Shao et al., 2007), to perform cluster analysis. The K-means identifies k number of centroids, and then allocates every data point to the nearest cluster, while maintaining the centroids as small as possible (Shao et al., 2007). We set the sieve parameter to 10 to reduce the expense of generating the pair-wise distance matrix by using “total/10” frames for initial clustering. The sieved frames are then added to the initial clusters. This analysis was done for 100,000 snapshots from the last 500 ns of each simulation, considering only the backbone atoms.

### Interaction Energy Network

Networks of local interactions are intrinsically linked to the structural response of proteins to external factors (O’Rourke et al., 2016). For our purposes, Weighted Implementation of Suboptimal Path (WISP) (Van Wart et al., 2014) was utilized to analyze the allosteric network. This method enabled us to perform a dynamic network analysis to understand how the binding of a ligand in an allosteric cavity could affect another binding site. In particular, WISP relies on the dynamical interdependence among the protein residues. To this end, each amino acid is treated as a node, which was located at the residue center-of-mass, and the interdependence among nodes is represented as a connecting edge with an associated numeric value that reflects its strength. The interdependence is determined from an *NxN* matrix *C* (N is the number of nodes) with values corresponding to the weights of each edge, reflecting the correlated motion among node-node pairs. Finally, the weight between the edge that connects nodes i and j is expressed as *wij* = −log(|*Cij*|), so that highly correlated or anticorrelated motions are characterized by small values of $w_{ij}$ . This analysis was performed for the last 500 ns of the MD simulations.

## Data Availability

The datasets presented in this study can be found in online repositories. The names of the repository/repositories and accession number(s) can be found below: http://www.wwpdb.org/, 5UFU; http://www.wwpdb.org/, 6B2E; http://www.wwpdb.org/, 6B1U.
