# Supplementary material for: Elucidating the Activation Mechanism of AMPK by Direct Pan-Activator PF-739
Source: Front Mol Biosci. 2021 Nov 5;8:760026. doi: 10.3389/fmolb.2021.760026 (PMC8602109; doi:10.3389/fmolb.2021.760026)
Supplement: Supplementary file 1 [file DataSheet1.PDF]

Supplementary Material

1 Supplementary Figures and Tables

1.1 Supplementary Figures

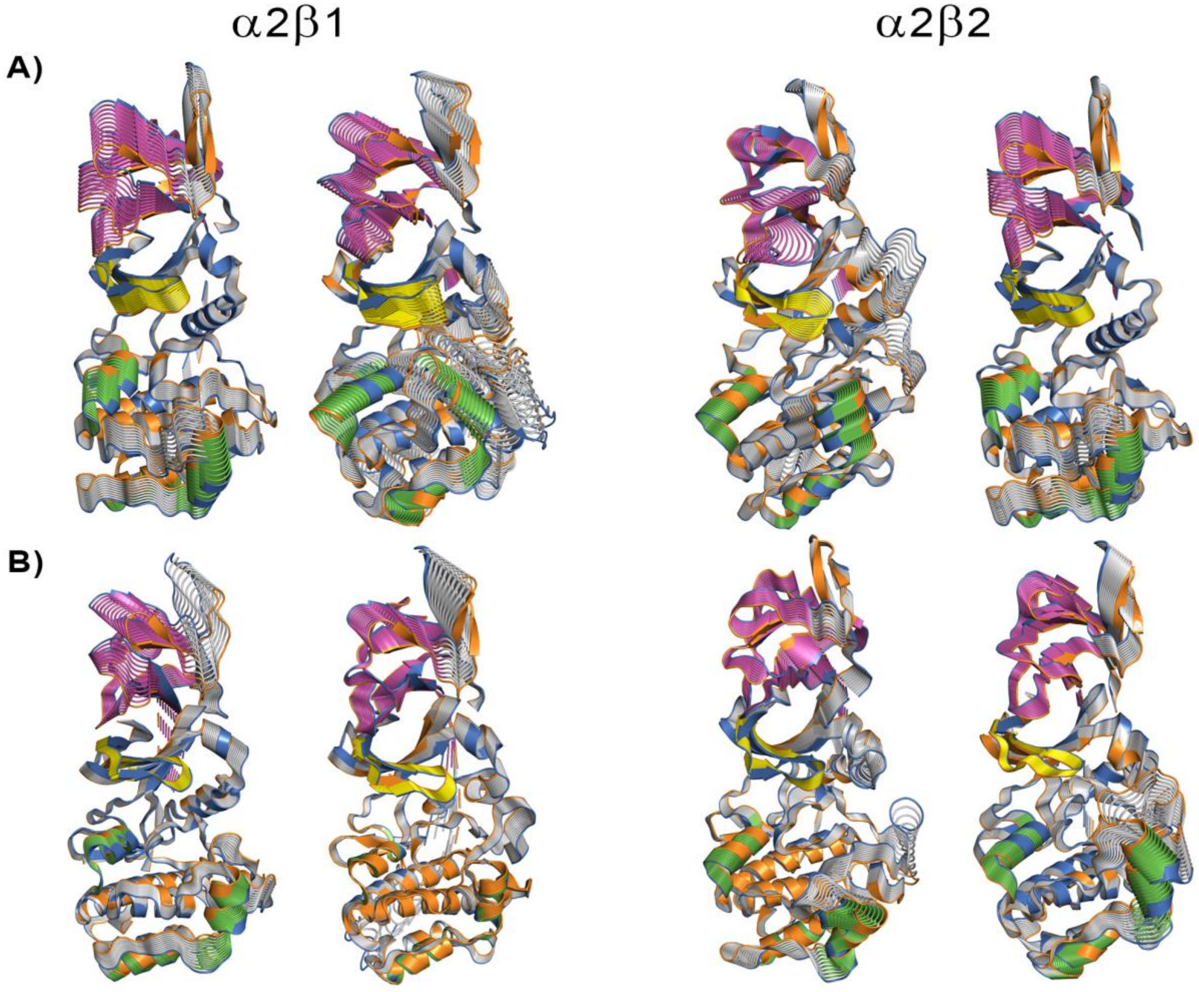

Supplementary Figure 1. Representation of the first essential motion obtained for two additional replicas run for A) holo and B) holo+ATP species PF-739 bound to α2β1 and α2β2 isoforms of AMPK. For the sake of clarity, the P-loop is shown in yellow, the helices formed by residues 100-110 and 220-229 in the α-subunit in green, and β-sheets in the CBM domain of the β-subunit in magenta.

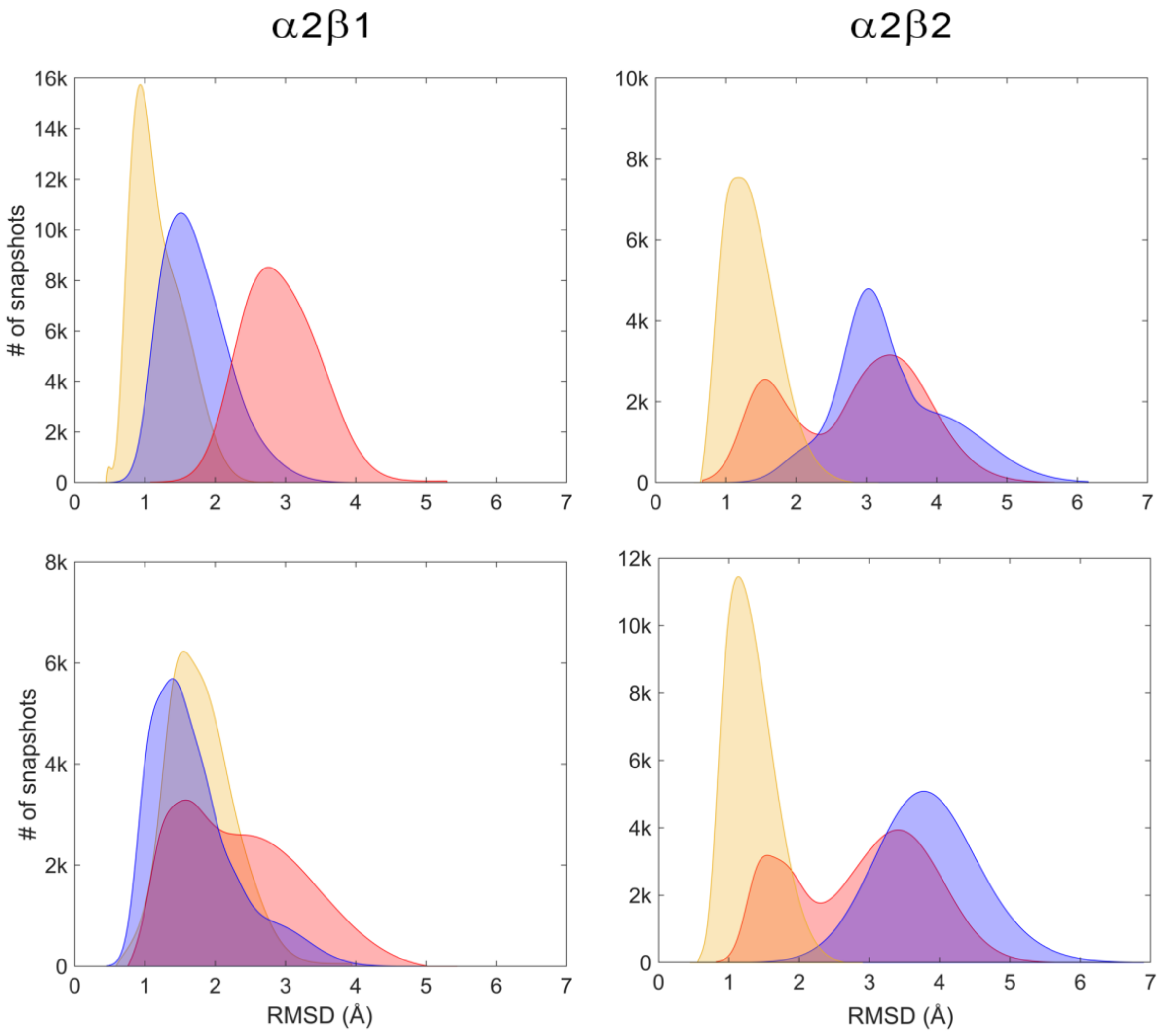

Supplementary Figure 2. Distribution of the positional deviation (RMSD; Å) of the structures sampled along the trajectories run for apo (red), holo (blue), and holo+ATP (orange) for the residues that shape the ATP-binding site (residues a22–a32, a42–a46, a75–a79, a142–a147, and a153–a157) in complexes bound to A-769662 (top) and SC4 (bottom). A total of 60,000 snapshots taken from the last 500 ns of MD simulations were considered for each system in the analysis.

1.2 Supplementary Tables

Supplementary Table 1. Similarity indexes determined for the the first 3 essential motions determined for the three independent simulations run for apo, holo and holo+ATP species of α2β1 and α2β2 isoforms.

Systems	α2β1[a]	α2β2[a]
**apo**	0.70 (0.07)	0.60 (0.09)
**holo**	0.75 (0.14)	0.47 (0.09)
**holo+ATP**	0.35 (0.02)	0.33 (0.04)

[a] Mean value of the three independent simulations and the standard deviation between parentheses.

Supplementary Table 2. Similarity indexes determined for the first 3 essential motions determined for the three independent simulations run for apo species of α2β1 and α2β2 isoforms.

Apo	α2β1	α2β2
**R1**	0.61	0.80
**R2**	0.81	0.81
**R3**	0.80	0.78

Supplementary Table 3. Similarity indexes determined for the first 3 essential motions using different time windows in replica 1 of apo - α2β1 system.

Apo-α2β1	R1
**From 200 to 600 ns + 600 to 1000 ns**	0.61
**From 350 to 650 ns + 650 to 1000 ns**	0.65
**From 400 to 700 ns + 700 to 1000 ns**	0.71

Supplementary Table 4. Similarity indexes determined for the dynamic cross-correlation (DCC) matrices obtained from the three independent simulations run for apo, holo and holo+ATP species of α2β1 and α2β2 isoforms. Values stand for the average similarity measured between each pair of replicas (standard deviation is given in parenthesis). The results are given for the comparison of dynamic correlation matrix of the whole protein (Global) and the distinct domains (α–α, β–β and α–β). Data obtained previously for activators A-769662 and SC4 are included for the sake of comparison.

**Species**	**α2β1**				**α2β2**			
	**Global**	**α–α**	**β–β**	**α–β**	**Global**	**α–α**	**β–β**	**α–β**
**apo**								
---	0.84 (0.02)	0.83 (0.02)	0.94 (0.02)	0.82 (0.02)	0.79 (0.06)	0.79 (0.08)	0.86 (0.03)	0.75 (0.05)
**holo**								
**A-769662**	0.91 (0.02)	0.92 (0.01)	0.94 (0.02)	0.89 (0.03)	0.75 (0.07)	0.80 (0.04)	0.83 (0.06)	0.59 (0.10)
**SC4**	0.84 (0.02)	0.84 (0.03)	0.89 (0.02)	0.83 (0.01)	0.75 (0.03)	0.79 (0.03)	0.76 (0.08)	0.66 (0.05)
**PF739**	0.66 (0.07)	0.64 (0.10)	0.84 (0.03)	0.63 (0.07)	0.66 (0.08)	0.71 (0.08)	0.72 (0.10)	0.55 (0.08)
**holo+ATP**								
**A-769662**	0.81 (0.01)	0.85 (0.01)	0.86 (0.05)	0.73 (0.03)	0.76 (0.02)	0.79 (0.03)	0.80 (0.03)	0.69 (0.04)
**SC4**	0.66 (0.10)	0.70 (0.12)	0.75 (0.03)	0.52 (0.10)	0.60 (0.05)	0.64 (0.06)	0.69 (0.14)	0.47 (0.06)
**PF739**	0.64 (0.07)	0.65 (0.08)	0.83 (0.02)	0.55 (0.06)	0.62 (0.12)	0.68 (0.08)	0.64 (0.20)	0.51 (0.14)

Supplementary Table 5. Distance measurements (Å) along the last 500 ns of the simulation of the holo state for α2β1 and α2β2 complexed with SC4 and PF-739, respectively. [a] Distance values (Å) for the time windows 200-600 ns and 600 – 1000 ns are reported, respectively, between parentheses. Each replica presents slightly different values due to the magnitude of the large-scale conformational motion.

Distance	Rep #1	Rep #2	Rep #3	Mean (SD)
**SC4@N4’···β1-N111**	8.4 ± 1.0 (8.3 ± 0.4/ 8.4 ± 1.1)	10.1 ± 1.3 (9.8 ± 1.4/ 10.2 ± 1.2)	8.8 ± 0.6 (8.6 ± 0.6/ 8.8 ± 0.6)	9.1 ± 0.9 (8.9 ± 0.8/ 9.1 ± 0.9)
**SC4@N4’···β2-D111**	8.1 ± 1.5 (9.1 ± 1.4/ 8.1 ± 1.6)	9.6 ± 1.1 (9.9 ± 1.1/ 9.5 ± 1.0)	9.8 ± 0.9 (9.9 ± 1.0/ 9.8 ± 0.9)	9.2 ± 1.2 (9.6 ± 1.1/ 9.1 ± 1.1)
**PF739@C4’···β1-N111**	9.5 ± 2.4 (9.3 ± 2.3/ 9.9 ± 2.3)	8.0 ± 2.6 (8.4 ± 2.1/ 8.1 ± 2.6)	10.3 ± 1.3 (9.4 ± 2.0/ 10.3 ± 1.3)	9.3 ± 2.1 (9.0 ± 2.1/ 9.4 ± 2.0)
**PF739@C4’···β2-D111**	9.0 ± 1.3 (9.8 ± 0.8/ 8.9 ± 1.5)	7.9 ± 1.1 (7.7 ± 1.1/ 7.9 ± 1.1)	9.8 ± 1.1 (10.0 ± 1.1/ 9.8 ± 1.1)	8.9 ± 1.2 (9.2 ± 1.0/ 8.8 ± 1.2)

Supplementary Table 6. Description of the systems used in Molecular Dynamics simulations.

**Systems**		**Protein**	**# Water molecules**	**# Na^+**	**# Cl^-**	**# Total Atoms**
**α2β1**	**apo**	α2: residues 8-278<br>4388 atoms	25,037	0	2	81,030
	**holo**	β1: residues 78-173<br>1526 atoms	26,641	0	0	85,897
	**holo+ATP**	PF-739:<br>54 atoms	26,622	4	0	85,887
**α2β2**	**apo**	α2: residues 8-278<br>4388 atoms	25,768	0	0	83,213
	**holo**	β2: residues 77-171<br>1518 atoms	25,718	2	0	83,122
	**holo+ATP**	PF-739:<br>54 atoms	25,725	6	0	83,190
